# Supplementary material for: Cotyledon opening during seedling deetiolation is determined by ABA-mediated splicing regulation
Source: EMBO Rep. 2025 Jun 18;26(14):3663–78. doi: 10.1038/s44319-025-00495-5 (PMC12287322; doi:10.1038/s44319-025-00495-5)
Supplement: Supplementary file 1 — Appendix [file 44319_2025_495_MOESM1_ESM.pdf]

Appendix

Cotyledon opening during seedling deetiolation is determined by ABA-mediated splicing regulation

Guiomar Martín, Ana Confraria, Irene Zapata, Alvaro Santiago Larran, Julia Irene Qüesta, Paula Duque

Table of Contents

Appendix Figure S1……………………………………………………………..............2
Appendix Figure S2……………………………………………………………..............3
Appendix Figure S3……………………………………………………………......4
Appendix Figure S3……………………………………………………………......5
Appendix Figure S4……………………………………………………………......6
Appendix Figure S5……………………………………………………………......7
Appendix Figure S7……………………………………………………………......8
Appendix Figure S8……………………………………………………………......9
Appendix Figure S9…………………………………………………………….....10
Appendix Figure S10…………………..………………………………………....11
Appendix Figure S11…………………..………………………………………....12
Appendix Figure S12…………………..………………………………………....13
Appendix Figure S13…………………..………………………………………....14
Appendix Figure S14…………………..………………………………………....15
Appendix Figure S15…………………..………………………………………....16
Appendix Figure S16…………………..………………………………………....17
Appendix Figure S17…………………..………………………………………....18
Appendix Figure S18…………………..………………………………………....19
Appendix Figure S19…………………..………………………………………....20
References……………………..………………..…………………………………....21

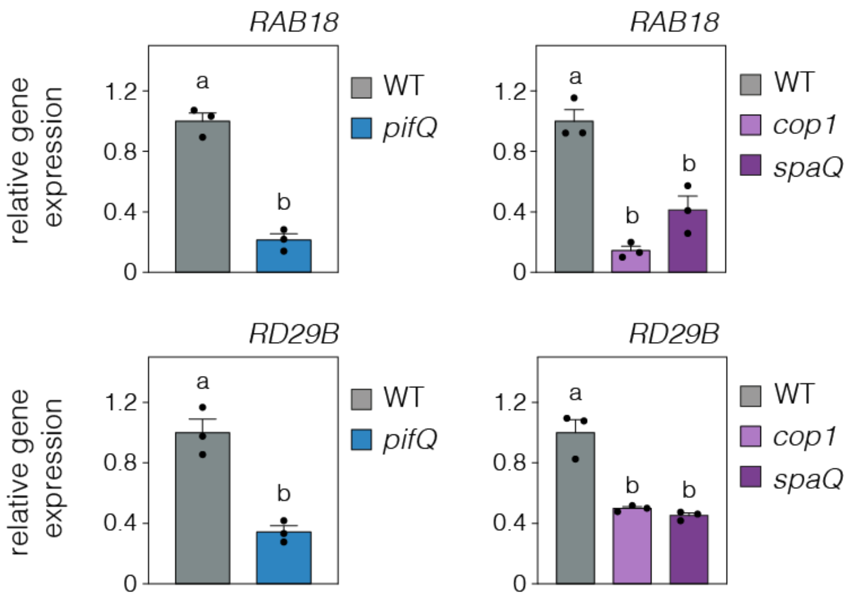

Appendix Figure S1. *RAB18* and *RD29B* expression levels in constitutive photomorphogenic mutants.

RAB18 and *RD29B* transcript levels, quantified from publicly available RNA sequencing data, of *pifQ*, *cop1* and *spaQ* seedlings and their respective wild type (WT) grown for 3 days in the dark. Data are the means ± SEM of biological triplicates, with different letters indicating statistically significant differences between genotypes ($P < 0.05$) by unpaired *t* test (left) and Tukey's multiple comparison test (right). RNA sequencing data were obtained from GSE112662 (Data ref: Pham et al., 2018) and GSE164122 (Data ref: Martín and Duque, 2021).

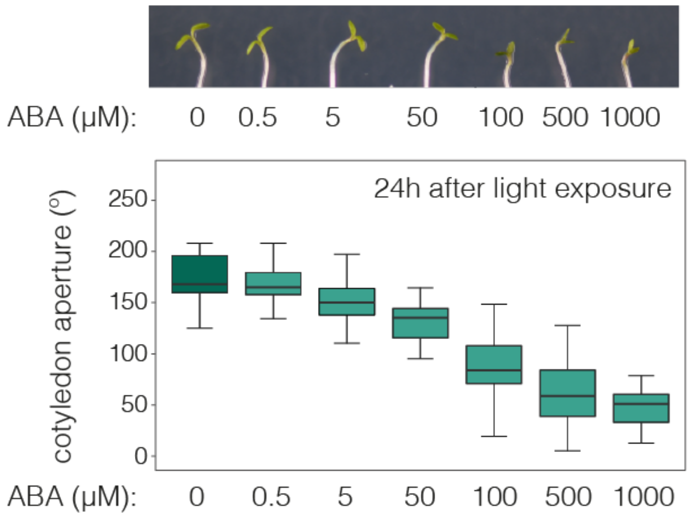

Appendix Figure S2. ABA repression of cotyledon aperture in the light. Representative image (top) and quantification of the cotyledon aperture (bottom) in at least 35 wild-type seedlings grown for 3 days in the dark and then exposed to white light in the presence of different concentrations of ABA. Cotyledon aperture is shown as the difference between the cotyledon angle of each seedling at 24 hours of ABA treatment and the median of the cotyledon angle at time 0 hours. Boxplots indicate the median (center line), interquartile range (box limits), and minimum and maximum values (whiskers). At least two biological replicates were conducted, all showing similar results.

A

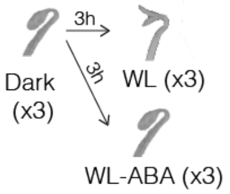

Name	Tissue	Pre-treatment	Treatment (3h)	
			Light	ABA
Dark	WT cotyledons	3-day-old dark grown seedlings	-	-
WL			45 mmol · m^{-2} · s^{-1}	0µM
WL-ABA			45 mmol · m^{-2} · s^{-1}	100µM

B

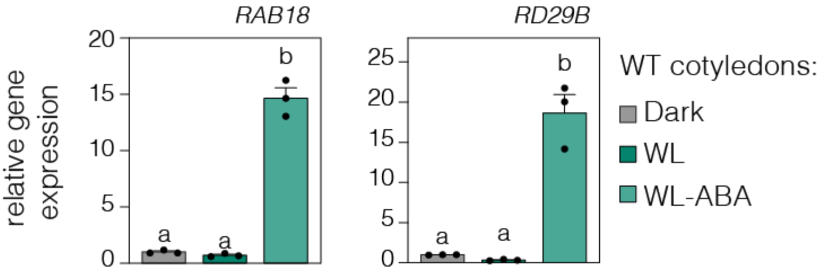

Appendix Figure S3. RNA-sequencing of ABA-treated cotyledons during seedling deetiolation.

(A) Schematic representation of the RNA-sequencing experimental design (left) and a summary table (right) outlining the technical details of each sample, sequenced in biological triplicates. **(B)** *RAB18* and *RD29B* transcript levels, quantified from our RNA sequencing data, in wild-type (WT) cotyledons from seedlings grown for 3 days in the dark and then exposed to continuous white light (WL) for 3 hours in the absence or presence of ABA (100 µM). Data are the means ± SEM of biological triplicates and relative to the dark timepoint. Different letters indicate statistically significant differences between conditions by Tukey's multiple comparison test ($P < 0.05$).

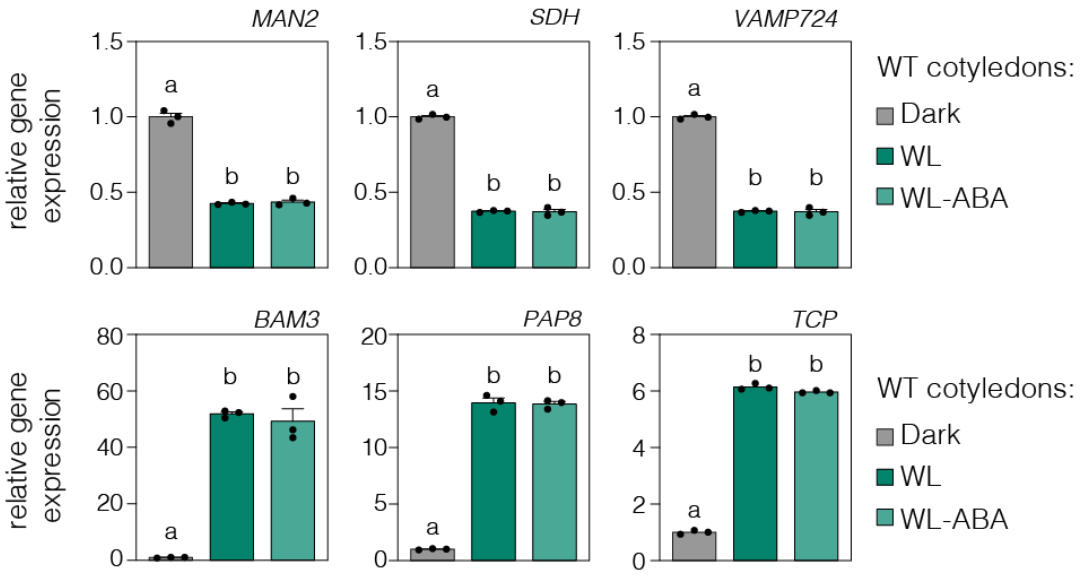

Appendix Figure S4. Expression levels of genes whose light responsiveness is unaffected by ABA.

Transcript levels, quantified from our RNA sequencing data, in wild-type (WT) cotyledons from seedlings grown for 3 days in the dark and then exposed to continuous white light (WL) for 3 hours in the absence or presence of ABA (100 µM). Data are the means ± SEM of biological triplicates and relative to the dark timepoint. Different letters indicate statistically significant differences between conditions by Tukey's multiple comparison test ($P < 0.05$).

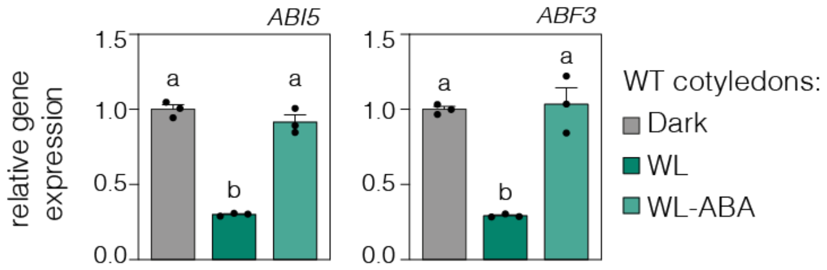

Appendix Figure S5. *ABI5* and *ABF3* expression levels in ABA-treated cotyledons during seedling deetiolation.

ABI5 and *ABF3* transcript levels, quantified from our RNA sequencing data, in wild-type (WT) cotyledons from seedlings grown for 3 days in the dark and then exposed to continuous white light (WL) for 3 hours in the absence or presence of ABA (100 μM). Data are the means ± SEM of biological triplicates and relative to the dark timepoint. Different letters indicate statistically significant differences between conditions by Tukey's multiple comparison test ($P < 0.05$).

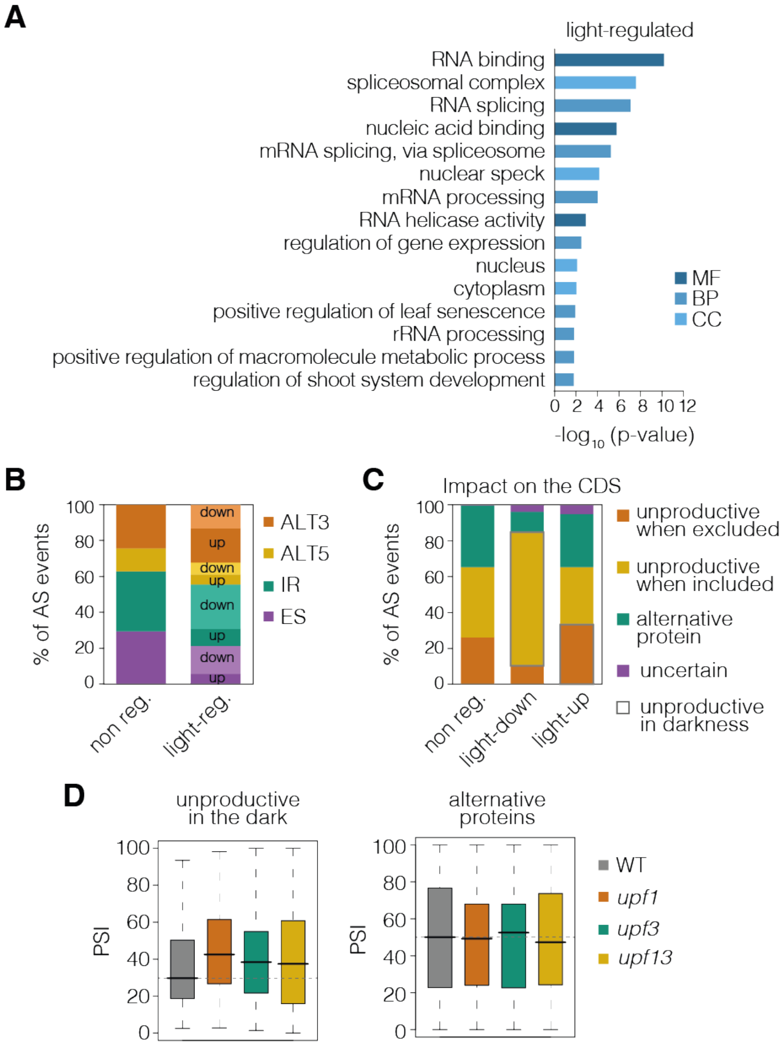

Appendix Figure S6. Light-regulated splicing changes in cotyledons from etiolated seedlings.

(A) Top 15 enriched gene ontology categories of molecular function (MF), biological process (BP) and cellular component (CC) for the 224 genes defined as differentially spliced in response to white light in cotyledons. DAVID *p*-value indicates significance (Fisher's exact test; $P < 0.05$; Dataset EV4). **(B)** Number of events by AS type for which the inclusion of the alternative sequence is up- or downregulated by light in comparison to non-regulated AS events (non reg.). ALT5, alternative 5´splice site; ALT3, alternative 3´splice site; IR, intron retention; ES, exon skipping. **(C)** Percentage of AS events located in gene coding sequence (CDS) regions that potentially generate unproductive mRNAs or alternative protein isoforms (see Methods for details) in three groups of AS events: non-regulated (non-reg.), up-, or downregulated by light. AS events that ultimately generate unproductive isoforms in the dark are indicated as "unproductive in darkness". **(D)** Percent of inclusion (PSI) values for the CDS-located light-regulated AS events that generate unproductive transcripts in the dark (left) or alternative proteins (right) in wild-type (WT), *upf1*, *upf3* and *upf1upf3* seedling samples. Boxplots indicate the median (center line), interquartile range (box limits), and minimum and maximum values (whiskers). This quantification was conducted with RNA sequencing data obtained from GSE41432 (Data ref: Drechsel et al., 2013).

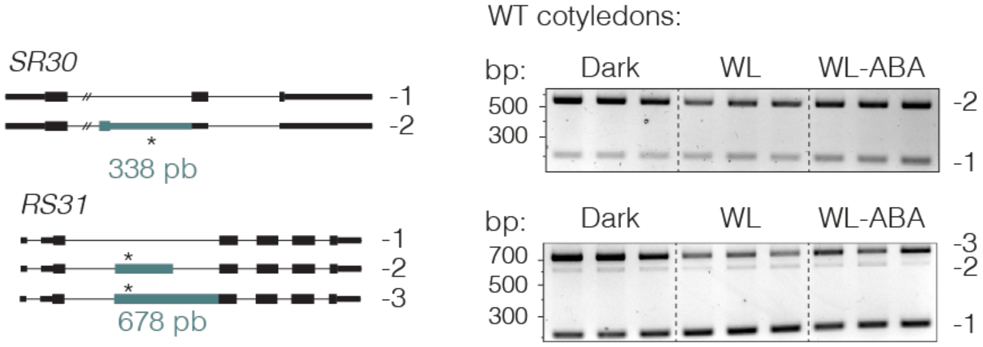

Appendix Figure S7. Light and ABA regulation of AS events in *SR30* and *RS31*. RT-PCR analysis of *SR30* (top) and *RS31* (bottom) alternative transcript levels in wild-type (WT) cotyledons from seedlings grown for 3 days in the dark and then exposed to continuous white light (WL) for 8 hours. The three gel lanes for each condition are biological triplicates, and the gene diagrams on the left show the alternative sequences in green, with asterisks indicating the location of in-frame premature stop codons. bp, base pairs.

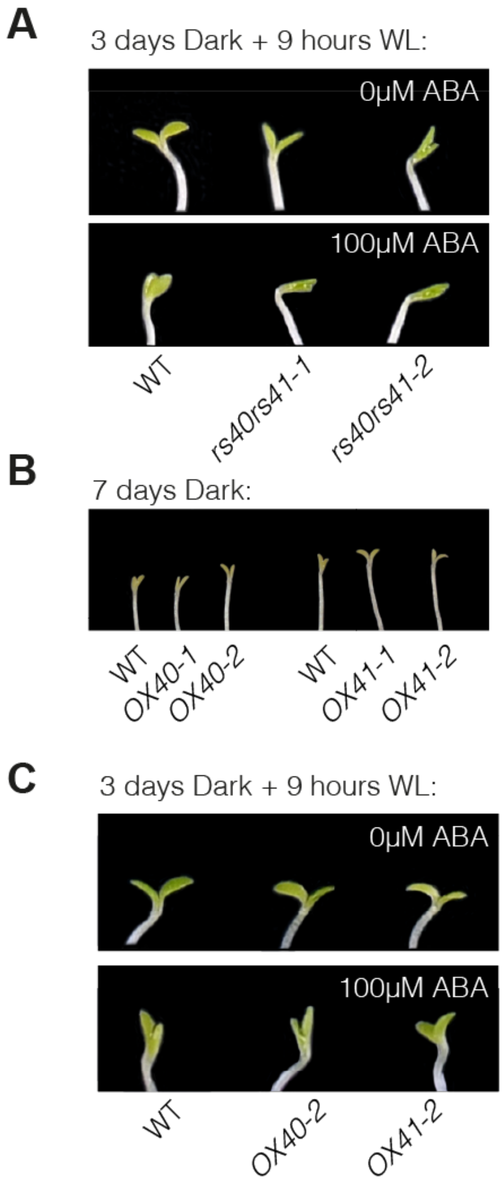

Appendix Figure S8. ABA regulation of light-induced cotyledon opening in *RS40* and *RS41* loss- and gain-of-function mutants.

Representative images of wild-type (WT) and *rs40 rs41* double mutant (**A**) or *RS40*- or *RS41*-overexpressing (**B-C**) seedlings, grown for 7 days in the dark (**B**) or for 3 days in the dark and then exposed to white light (WL) for 9 hours in the presence or absence of ABA (**A, C**).

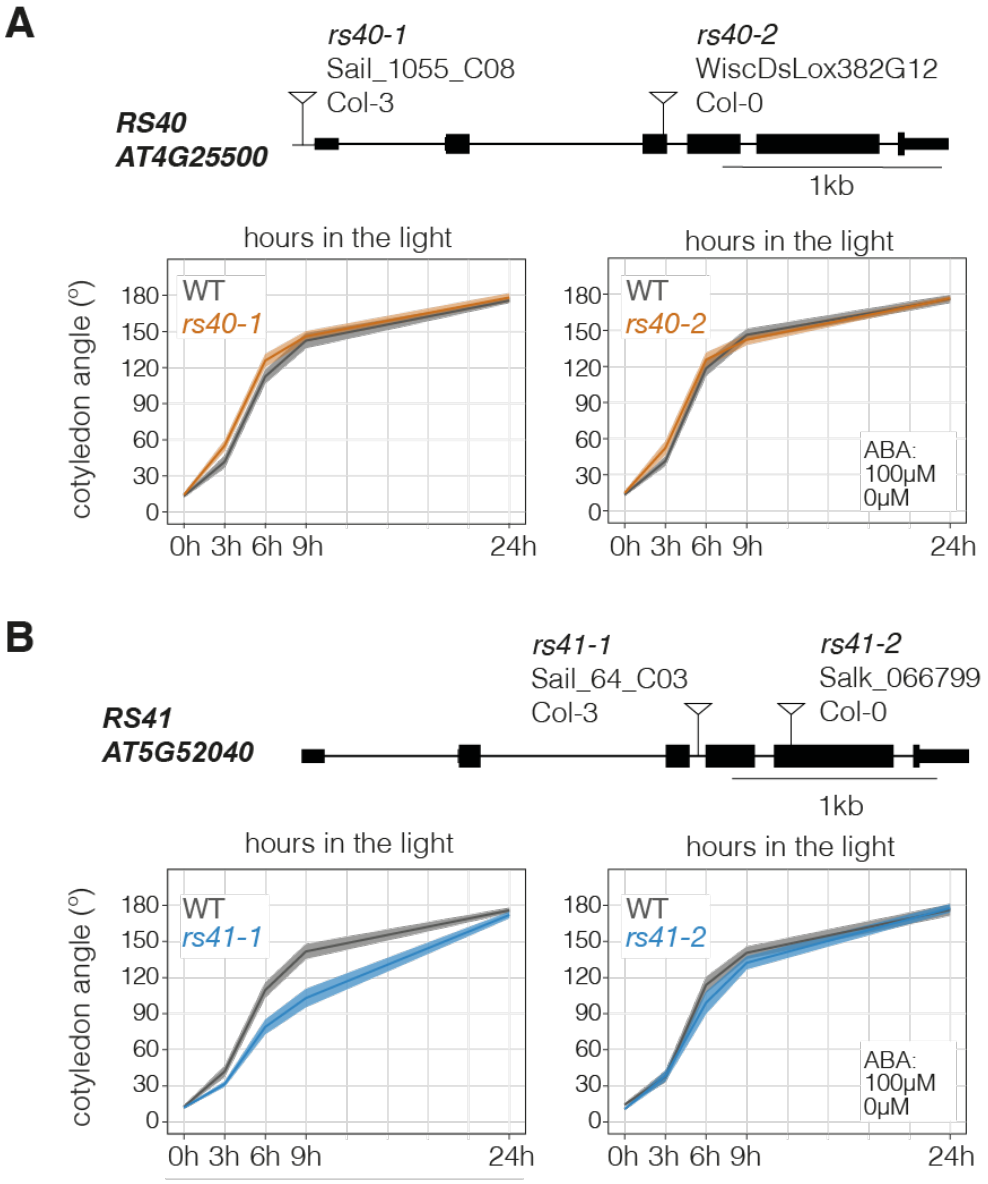

Appendix Figure S9. Light regulation of cotyledon opening in *rs40* and *rs41* single mutants.

For *RS40* **(A)** and *RS41* **(B),** gene diagrams (top) indicating the T-DNA insertion sites for two mutant lines per gene and the corresponding cotyledon phenotypes (bottom) are shown. Quantification of cotyledon opening was conducted in each mutant and its respective wild-type (WT) background (Col-0 or Col-3). Seedlings were grown for 3 days in the dark and then exposed to white light for 3, 6, 9 or 24 hours (h). Thick lines and shaded areas represent respectively the median and the 95% confidence interval of at least 45 seedlings. At least two biological replicates were conducted, all showing similar results.

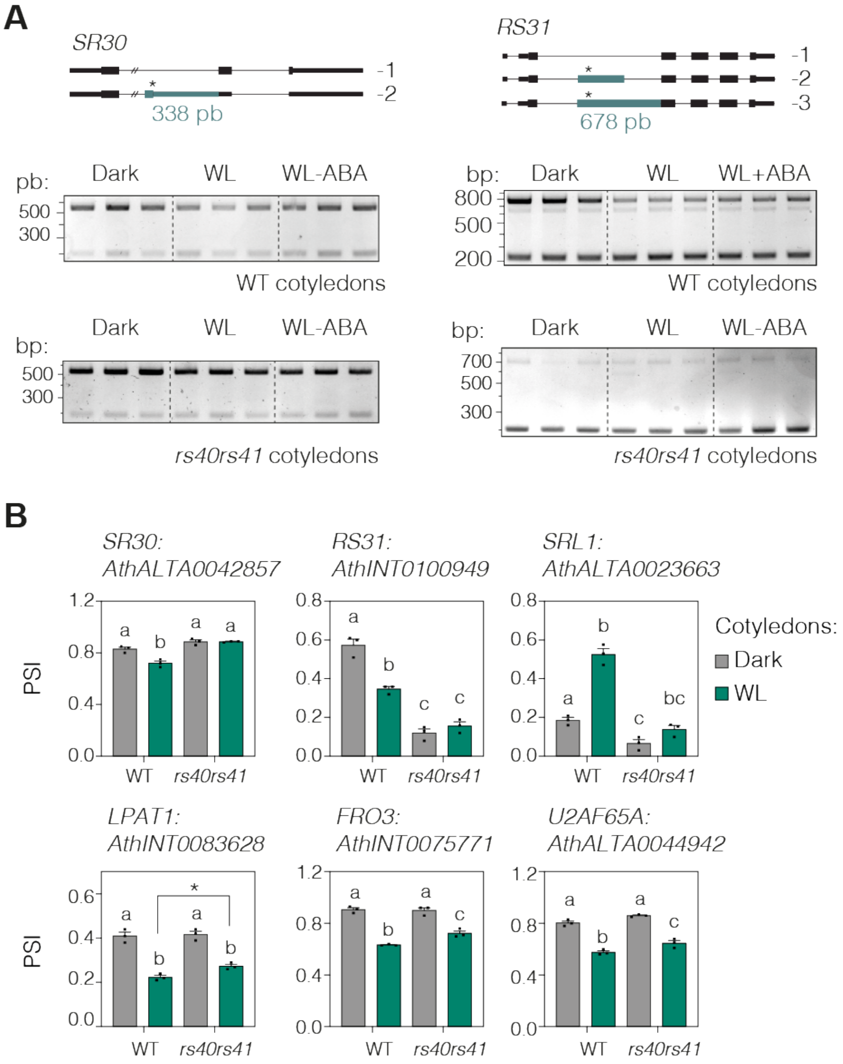

Appendix Figure S10. Light regulation of AS in the *rs40 rs41* double mutant.
(A) RT-PCR analysis of *SR30* and *RS31* alternative transcript levels in wild-type (WT) and *rs40rs41-1* cotyledons of seedlings grown for 3 days in the dark and then exposed to continuous white light (WL) for 8 hours in the absence or presence of ABA (100 µM). The three gel lanes for each condition are biological triplicates, and the gene diagrams show the alternative sequences in green, with asterisks indicating the location of in-frame premature stop codons. bp, base pairs. **(B)** RT-PCR quantification of light-regulated alternative splicing event levels in seedlings grown in the dark or light as indicated in (A). The identity of each splicing event is based on the information available in PastDB (Plant alternative splicing and transcription Data Base; www.pastdb.crg.eu). The bar graphs present Percent Splice In (PSI) values after quantification of the band intensities using the Image J software. Data are the means ± SEM of biological triplicates. Different letters indicate statistically significant differences between conditions by Tukey's multiple comparison test ($P < 0.05$) and the asterisk indicates statistically significant differences between genotypes under WL conditions (Unpaired *t* test; *, $P = 0.0160$).

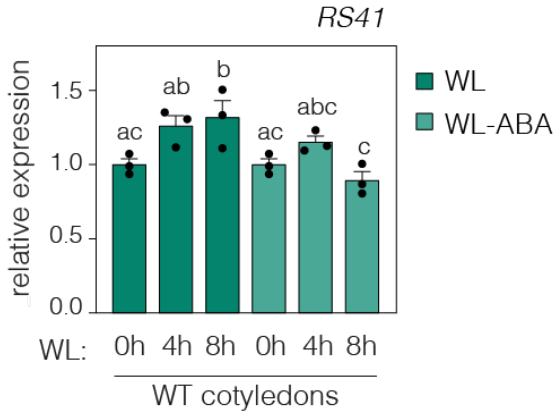

Appendix Figure S11. ABA regulation of *RS41* expression during seedling deetiolation.

RT-qPCR analysis of *RS41* transcript levels in cotyledons of wild-type (WT) seedlings grown for 3 days in the dark and then exposed to continuous white light (WL) for 4 or 8 hours (h) in the absence or presence of ABA (100 µM). *PP2A* was used as a reference gene, and expression levels in the dark-grown (0h WL) were set to 1. Data are the means ± SEM of biological triplicates, and different letters indicate statistically significant differences between conditions by Tukey's multiple comparison test ($P < 0.05$).

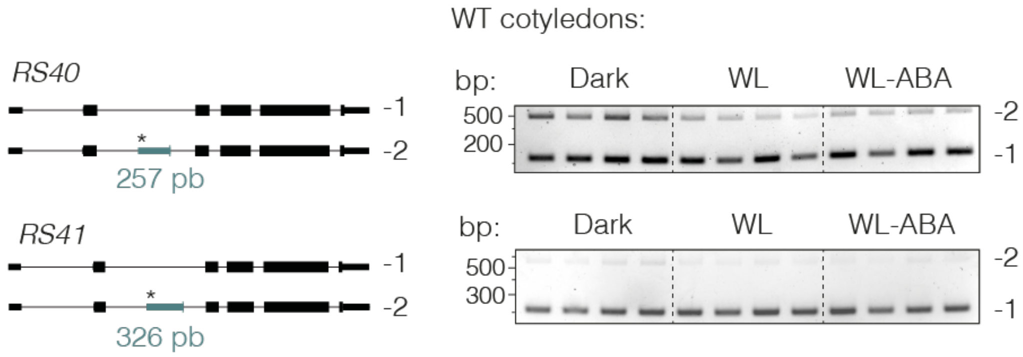

Appendix Figure S12. Light and ABA regulation of AS events in *RS40* and *RS41*. RT-PCR analysis of *RS40* (top) and *RS41* (bottom) alternative transcript levels in wild-type (WT) cotyledons of seedlings grown for 3 days in the dark and then exposed to continuous white light (WL) for 3 hours in the absence or presence of ABA (100 µM). The four gel lanes for each condition are biological quadruplets, and the gene diagrams on the left show the alternative sequences in green, with asterisks indicating the location of in-frame premature stop codons. bp, base pairs.

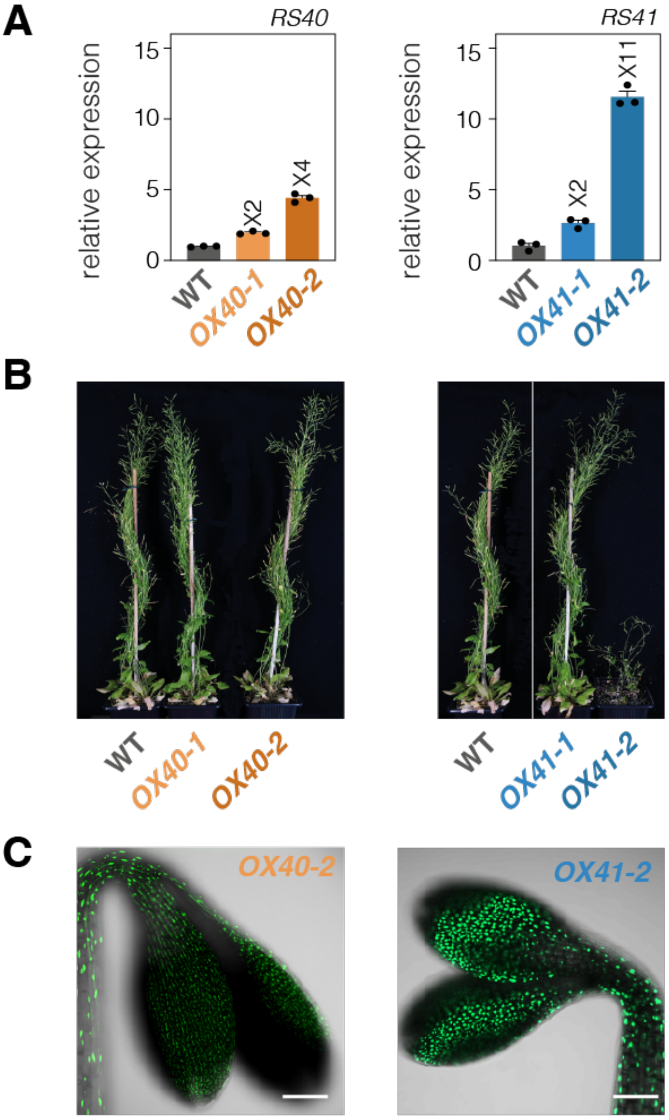

Appendix Figure S13. Generation of transgenic plants overexpressing *RS40* and *RS41*.

(A) RT-qPCR analysis of *RS40* and *RS41* transcript levels in the wild-type (WT) and two transgenic overexpression lines grown for 3 days in the dark and then exposed to continuous white light for 6 hours. *PP2A* was used as a reference gene, and expression levels in the WT were set to 1. Data are the means ± SEM of technical triplicates. **(B)** Representative images of adult plants of each transgenic line and a representative WT plant grown in parallel. **(C)** Confocal laser scanning microscopy images of the RS40-GFP and RS41-GFP proteins from transgenic plants grown in the dark for 3 days. Scale bar, 200 µm.

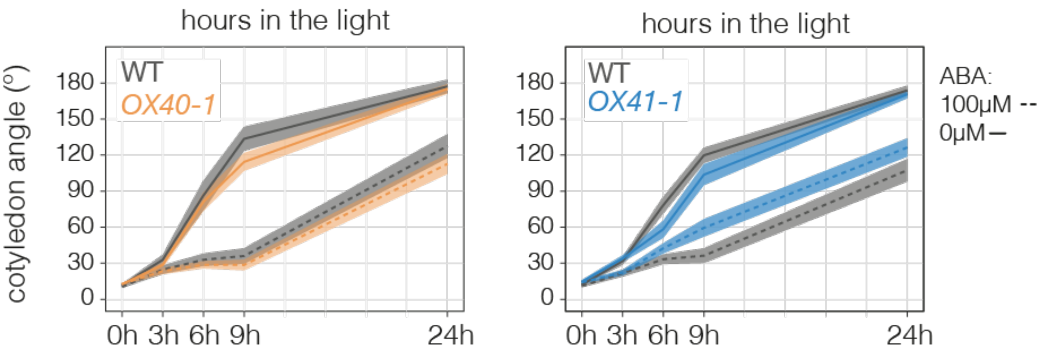

Appendix Figure S14. Light and ABA regulation of cotyledon opening in plants overexpressing *RS40* or *RS41*.

Quantification of cotyledon opening in the wild-type (WT) and *RS40* or *RS41* transgenic seedlings grown for 3 days in the dark and then exposed to white light for 3, 6, 9 or 24 hours (h) in the absence or presence of ABA. Thick lines and shaded areas represent respectively the median and the 95% confidence interval of at least 70 seedlings. At least two biological replicates were conducted, all showing similar results.

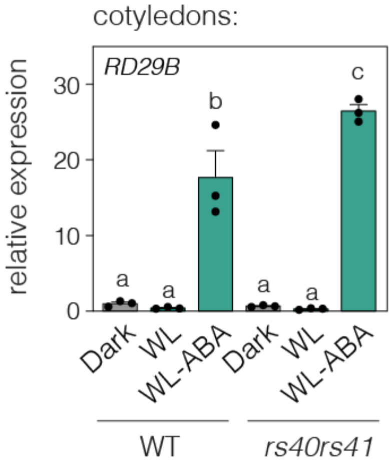

Appendix Figure S15. *RD29B* expression levels in ABA-treated *rs40 rs41* double mutants.
RT-qPCR analysis of *RD29B* transcript levels in wild-type (WT; Col-3) and *rs40rs41-1* cotyledons of seedlings grown for 3 days in the dark and then exposed to continuous white light (WL) for 8 hours in the absence or presence of ABA (100 µM). *PP2A* was used as a reference gene, and expression levels in the WT were set to 1. Data are the means ± SEM of biological triplicates, and different letters indicate statistically significant differences between conditions by Tukey's multiple comparison test (P < 0.05).

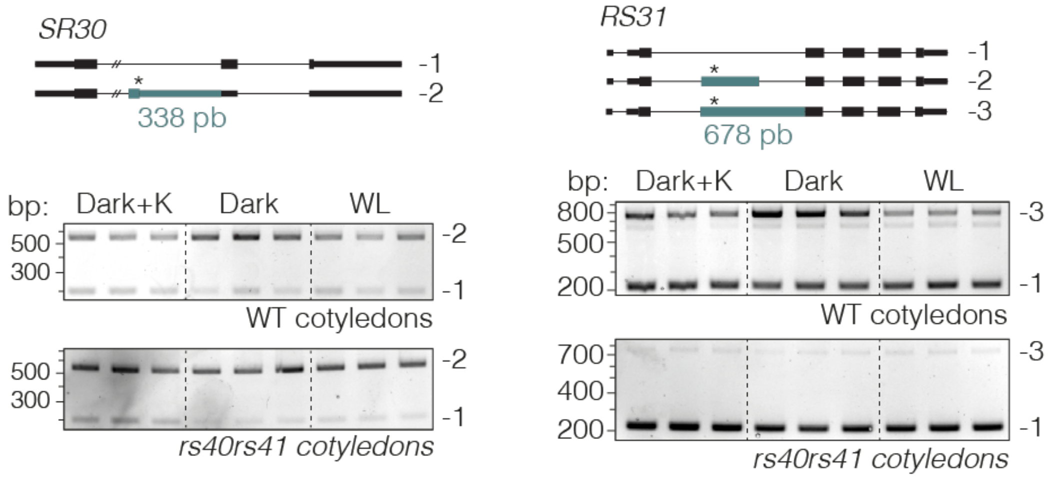

Appendix Figure S16. K252a regulation of *SR30* and *RS31* light- and ABA-regulated AS events.

RT-PCR analysis of *SR30* (left) and *RS31* (right) alternative transcripts in wild-type (WT; Col-3) and *rs40rs41-1* cotyledons of seedlings grown for 3 days in the dark and then exposed to continuous white light (WL) or the kinase inhibitor K252a (K; 1 μM) for 8 hours in the dark. The three gel lanes for each condition are biological triplicates, and the gene diagrams on the top show the alternative sequences in green, with asterisks indicating the location of in-frame premature stop codons. Images of WT cotyledons under Dark and WL contconditions are reused from Appendix Fig. 10. bp, base pairs.

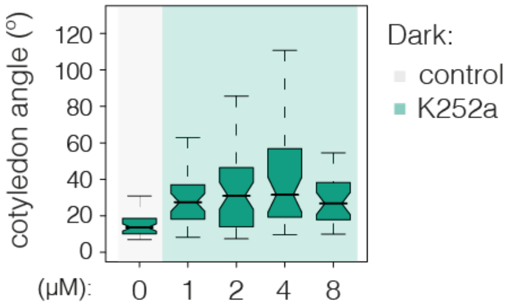

Appendix Fig. S17. K252a regulation of cotyledon opening in etiolated seedlings.
Quantification of cotyledon opening in wild-type seedlings grown for 3 days in the dark and then treated with different concentrations of the kinase inhibitor K252a for 9 hours. Boxplots indicate the median of at least 30 seedlings (center line), interquartile range (box limits), and minimum and maximum values (whiskers).

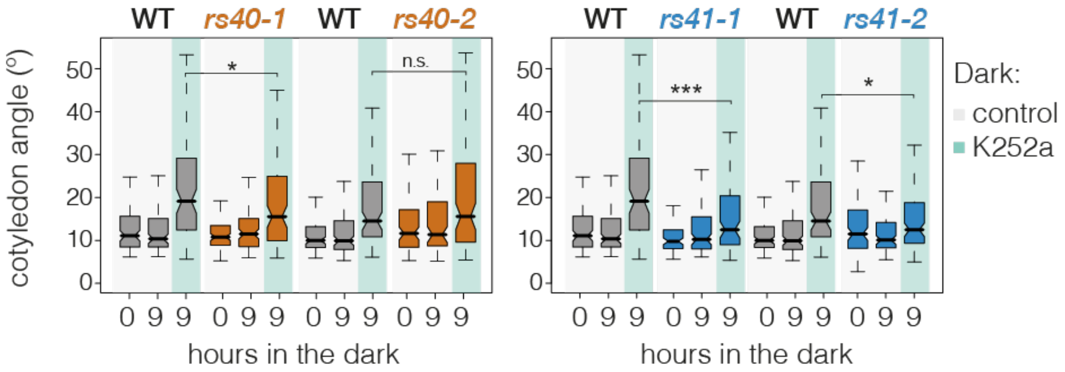

Appendix Figure S18. K252a regulation of cotyledon opening in the *rs40* and *rs41* single mutants.

Boxplot representation of cotyledon opening in the *rs40* (left) and *rs41* (right) single mutants and their respective wild-type (WT) seedlings (Col-0 or Col-3; see Methods for details) grown for 3 days in the dark and then treated or not with the kinase inhibitor K252a (1 μM) for 9 hours. Boxplots indicate the median of at least 80 seedlings (center line), interquartile range (box limits), and minimum and maximum values (whiskers). Asterisks indicate statistically significant differences between K252a-treated mutants and their respective WTs (Mann–Whitney test: WT vs. *rs40-1* $P = 0.0322$; WT vs. *rs40-2* $P = 0.997$; WT vs. *rs41-1* $P < 0.0001$; WT vs. *rs41-2* $P < 0.0392$). At least two biological replicates were conducted, all showing similar results.

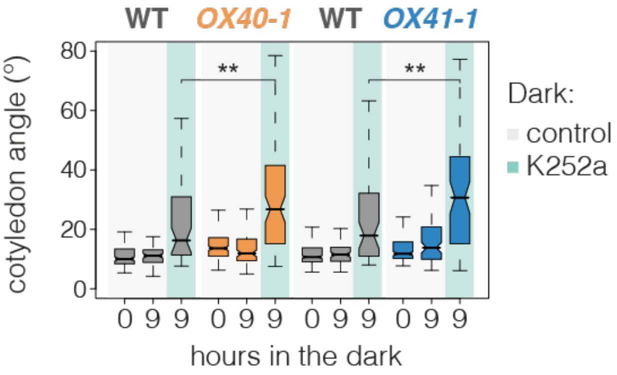

Appendix Figure S19. K252a regulation of cotyledon opening in plants overexpressing *RS40* or *RS41*.

Quantification of cotyledon opening in wild-type (WT) and *RS40*- or *RS41*-overexpressing seedlings grown for 3 days in the dark and then treated or not with the kinase inhibitor K252a (1 µM) for 9 hours. Boxplots indicate the median of at least 50 seedlings (center line), interquartile range (box limits), and minimum and maximum values (whiskers). Asterisks indicate statistically significant differences between treated transgenic plants and their respective WTs (Mann–Whitney test: WT vs. *OX40-1* P = 0.0088; WT vs. *OX41-1* P = 0.0032). At least two biological replicates were conducted, all showing similar results.

References

Drechsel, G., Kahles, A., Kesarwani, A. K., Stauffer, E., Behr, J., Drewe, P., Rätsch, G., and Wachter, A. (2013). Gene Expression Omnibus GSE41432 (https://www.ncbi.nlm.nih.gov/geo/query/acc.cgi?acc=GSE41432). [DATASET]

Drechsel, G., Kahles, A., Kesarwani, A. K., Stauffer, E., Behr, J., Drewe, P., Rätsch, G., and Wachter, A. (2013). Nonsense-mediated decay of alternative precursor mRNA splicing variants is a major determinant of the Arabidopsis steady state transcriptome. *Plant Cell* **25**:3726–3742.

Martín, G., and Duque, P. (2021). Gene Expression Omnibus GSE164122 (https://www.ncbi.nlm.nih.gov/geo/query/acc.cgi?acc=GSE164122). [DATASET]

Martín, G., and Duque, P. (2021). Tailoring photomorphogenic markers to organ growth dynamics. *Plant Physiol* **186**:239-249.

Pham, V. N., Xu, X., and Huq, E. (2018). Gene Expression Omnibus GSE112662 (https://www.ncbi.nlm.nih.gov/geo/query/acc.cgi?acc=GSE112662). [DATASET]

Pham, V. N., Xu, X., and Huq, E. (2018). Molecular bases for the constitutive photomorphogenic phenotypes in Arabidopsis. *Development* **145**:dev169870.
