# Supplementary figures and images for: Cotyledon opening during seedling deetiolation is determined by ABA-mediated splicing regulation
Source: EMBO Rep. 2025 Jun 18;26(14):3663–78. doi: 10.1038/s44319-025-00495-5 (PMC12287322; doi:10.1038/s44319-025-00495-5)

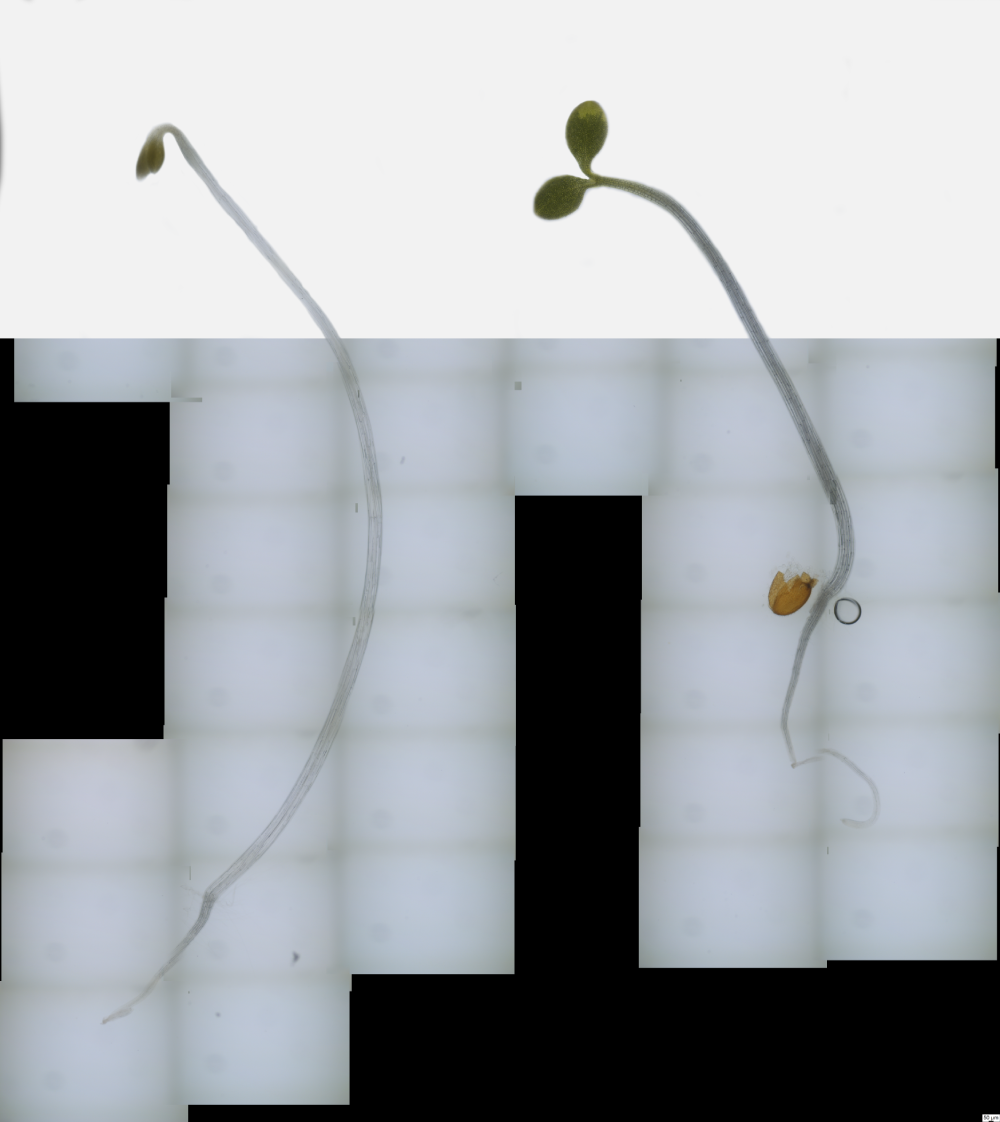

Supplement: Supplementary file 8 — Source data Fig. 1 [file 44319_2025_495_MOESM8_ESM.zip › Figure_1/1A/24hD_24hWL_35S GFP_BF.tiff]

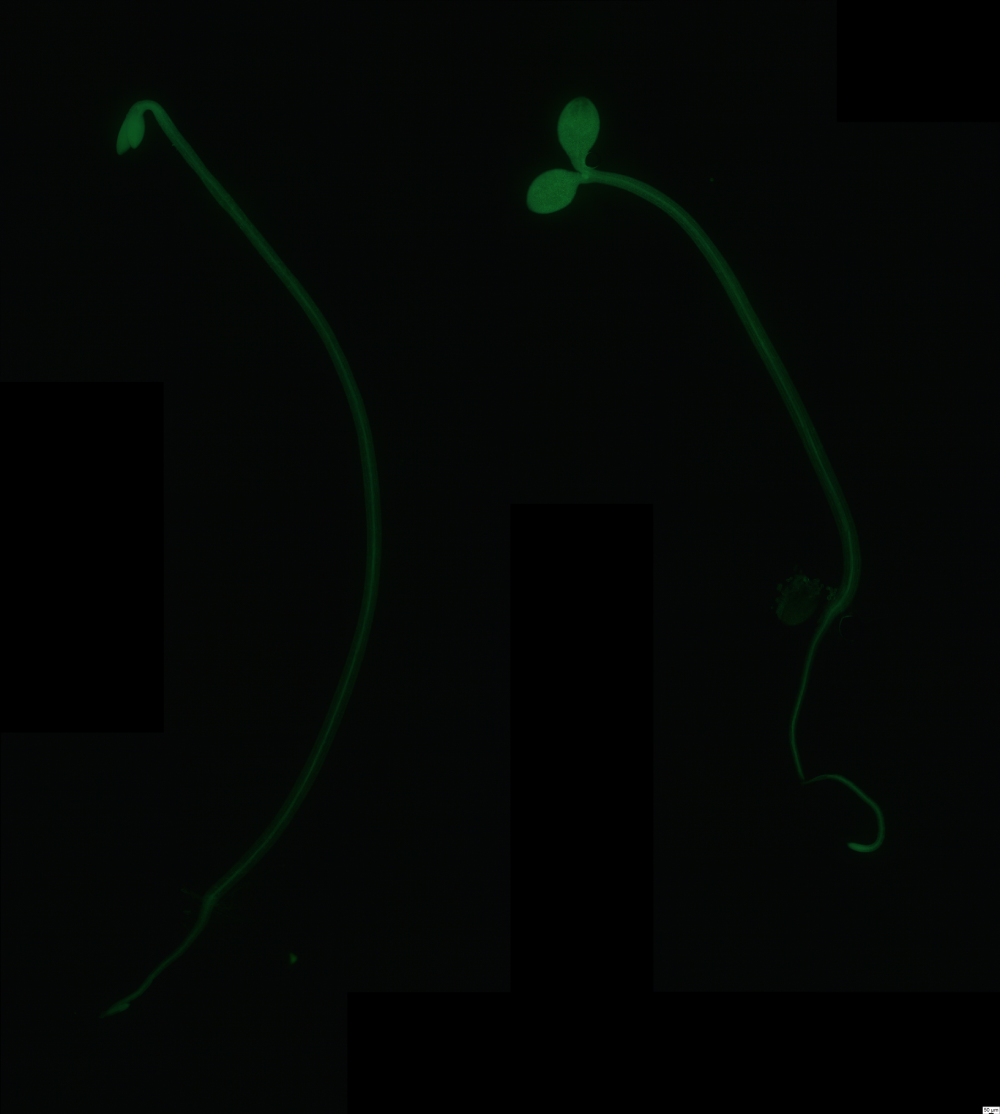

Supplement: Supplementary file 8 — Source data Fig. 1 [file 44319_2025_495_MOESM8_ESM.zip › Figure_1/1A/24hD_24hWL_35S GFP_GFP.tiff]

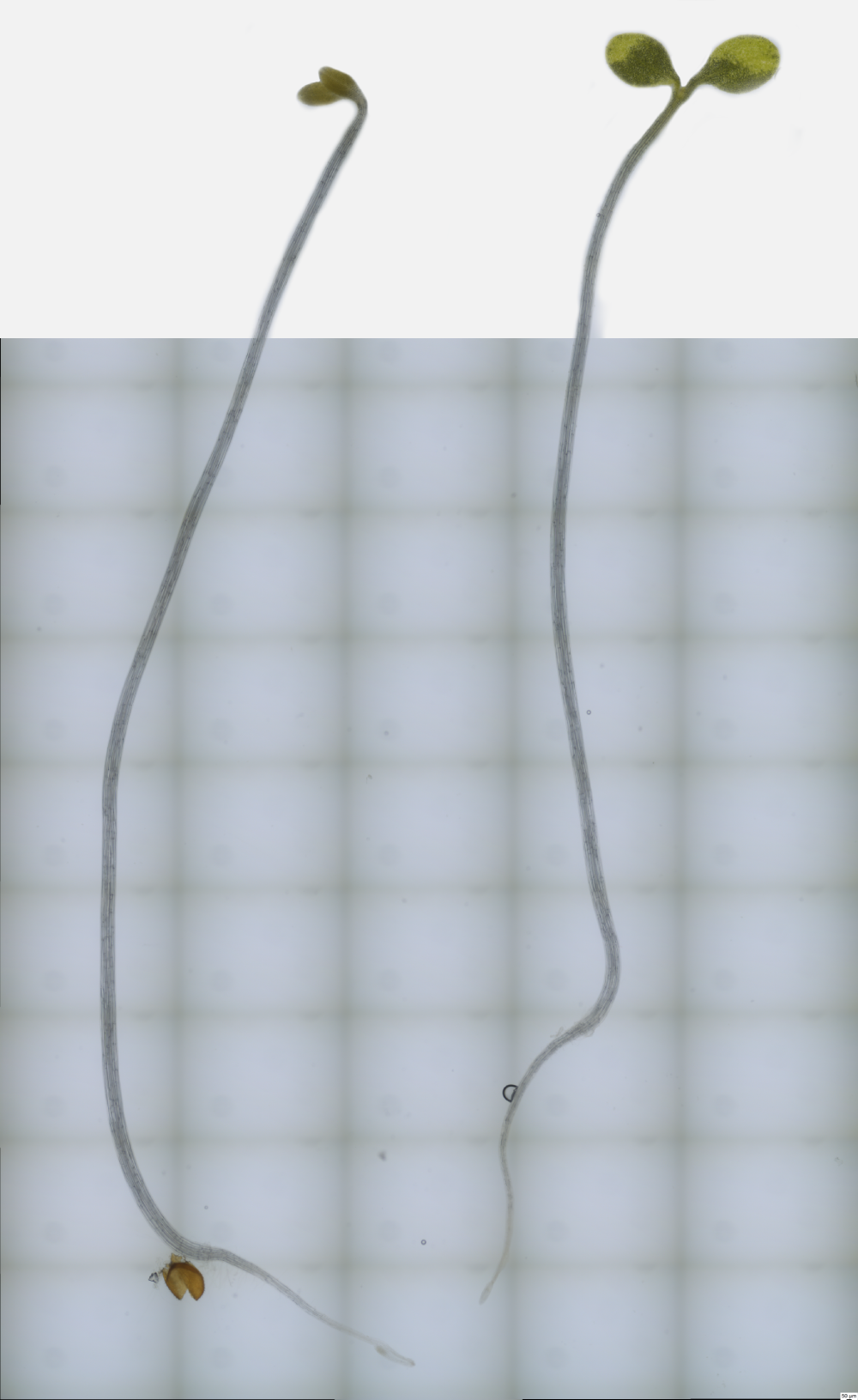

Supplement: Supplementary file 8 — Source data Fig. 1 [file 44319_2025_495_MOESM8_ESM.zip › Figure_1/1A/24hD_24hWL_RAB18_BF.tiff]

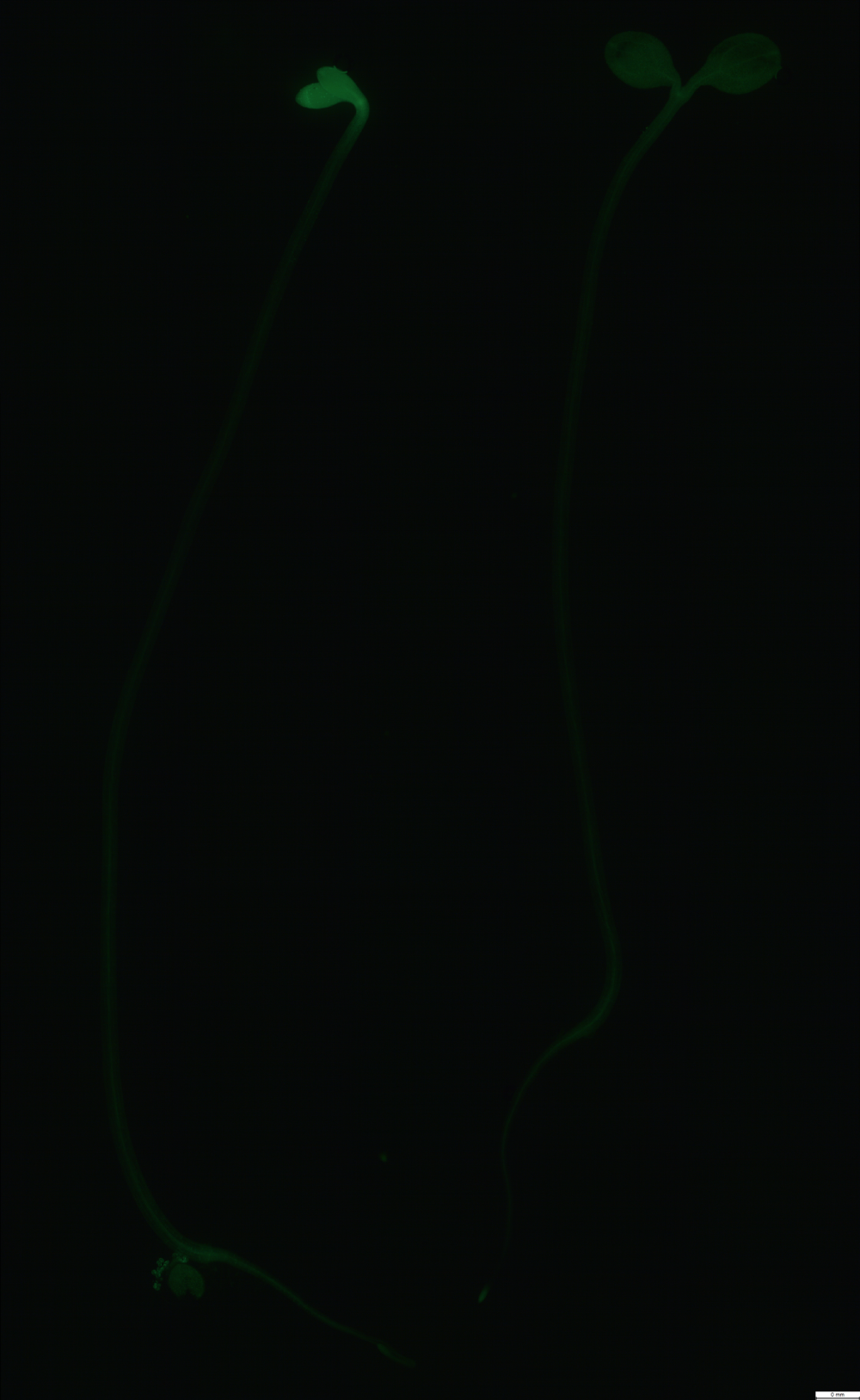

Supplement: Supplementary file 8 — Source data Fig. 1 [file 44319_2025_495_MOESM8_ESM.zip › Figure_1/1A/24hD_24hWL_RAB18_GFP.tiff]

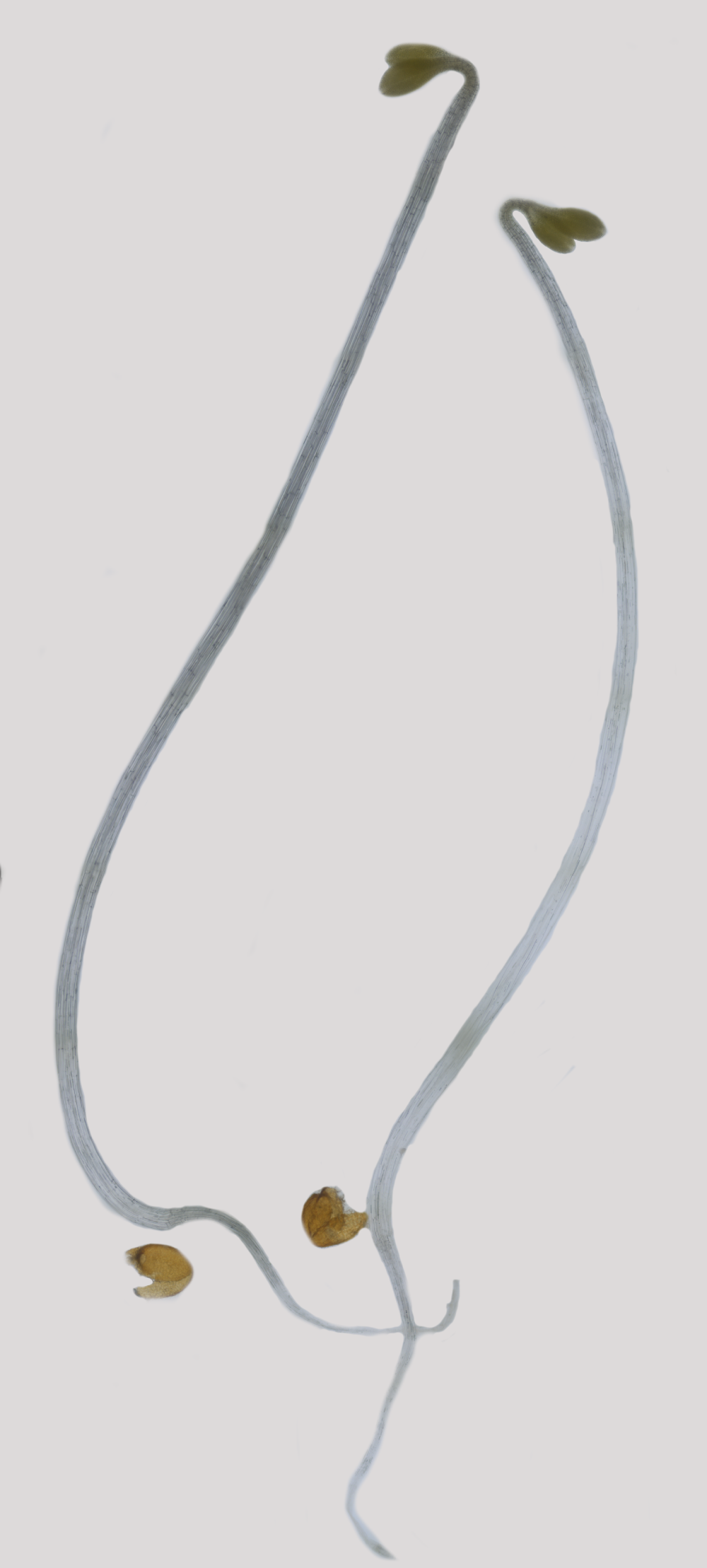

Supplement: Supplementary file 8 — Source data Fig. 1 [file 44319_2025_495_MOESM8_ESM.zip › Figure_1/1A/3 days Darkness_BF.tiff]

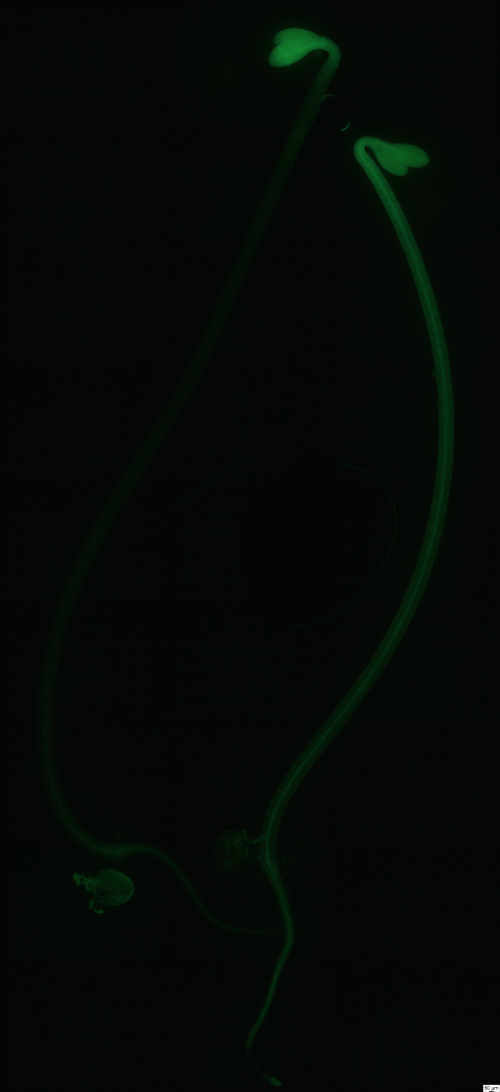

Supplement: Supplementary file 8 — Source data Fig. 1 [file 44319_2025_495_MOESM8_ESM.zip › Figure_1/1A/3 days Darkness_GFP.tiff]

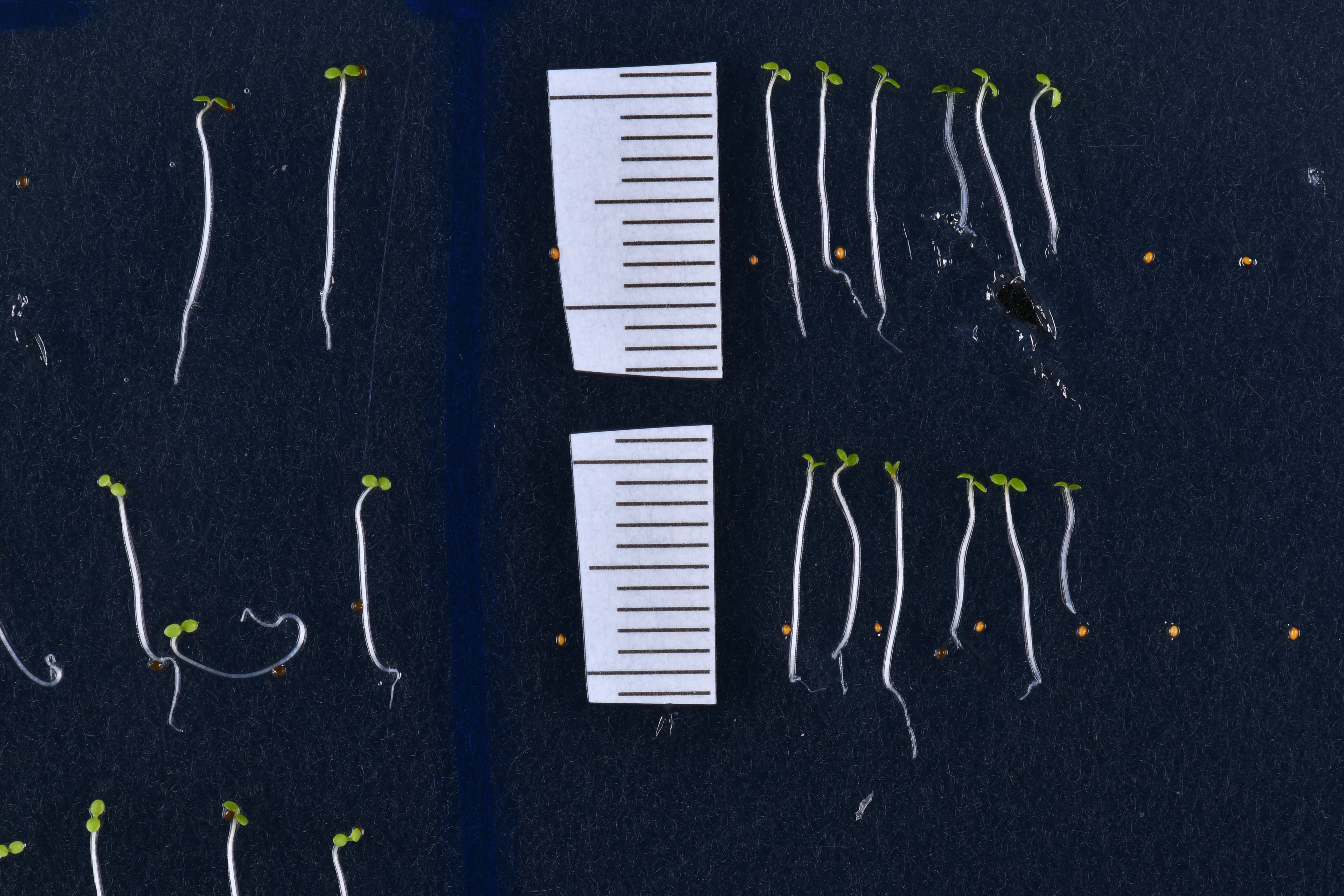

Supplement: Supplementary file 8 — Source data Fig. 1 [file 44319_2025_495_MOESM8_ESM.zip › Figure_1/1E/DSC_1792.JPG]

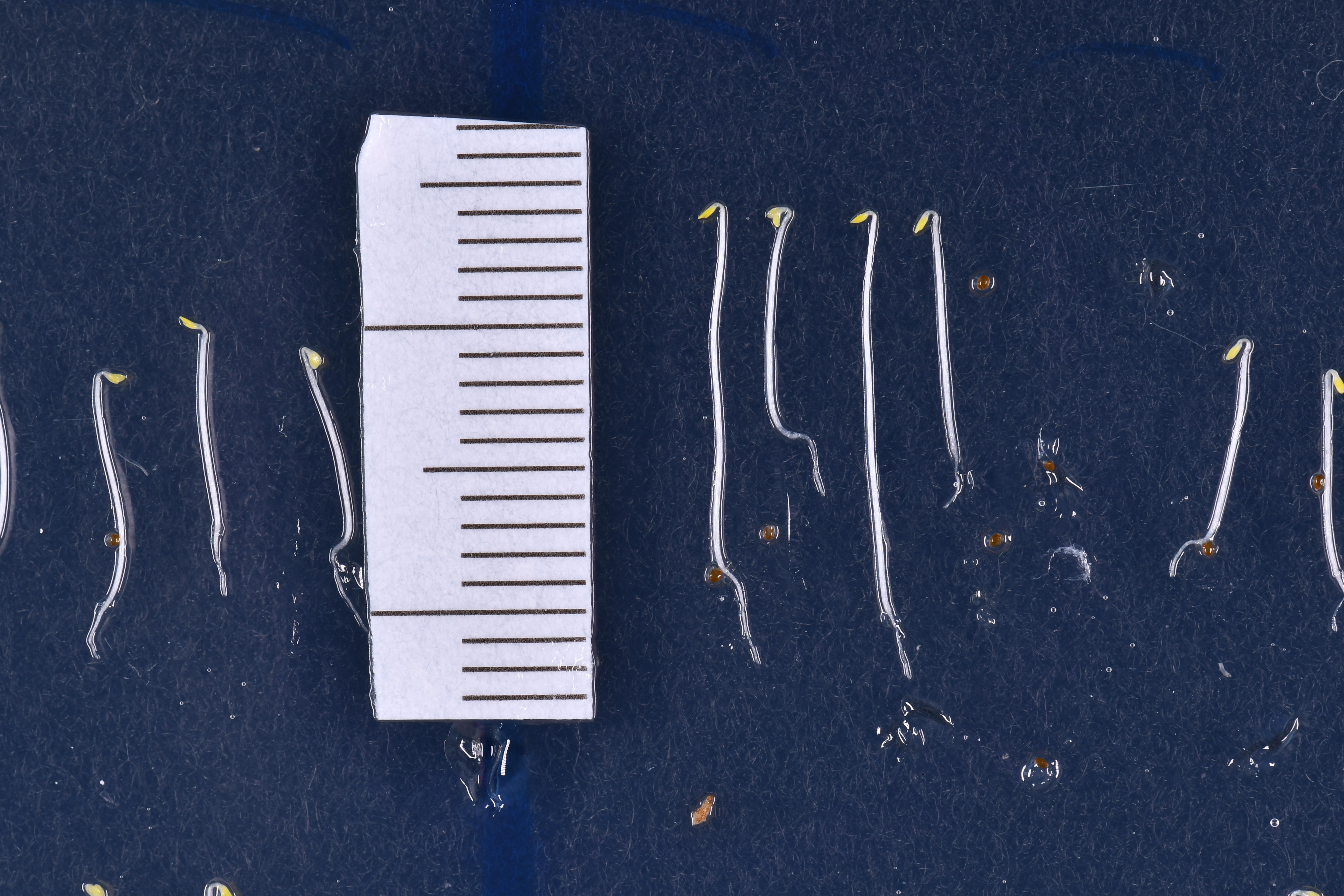

Supplement: Supplementary file 12 — Source data Fig. 5 [file 44319_2025_495_MOESM12_ESM.zip › Figure_5/5A/DSC_1112.JPG]
